# Targeting CD74 in microglia to modulate experimental cerebral ischemia and reperfusion injury: insights from Single-Cell and bulk transcriptomics

**DOI:** 10.1186/s13041-025-01197-8

**Published:** 2025-05-21

**Authors:** Chang Cao, Ting Liu, Lu Peng, Lianxin Li, Zhongmou Xu, Xiang Li, Gang Chen, Haiying Li, Lei Bai

**Affiliations:** 1 Department of Neurosurgery & Brain and Nerve Research Laboratory, The First Affiliated Hospital of Soochow University, Soochow University, Suzhou, 215006 China; 2 Institute of Stroke Research, Soochow University, Suzhou, 215006 China; 3 Department of Traditional Chinese Medicine, Shandong Provincial Hospital Affiliated to Shandong First Medical University, Jinan, China; 4 Department of Neurosurgery & Brain and Nerve Research Laboratory, The First Affiliated Hospital of Soochow University, Suzhou, Jiangsu Province China; 5 188 Shizi Street, Suzhou, 215006 Jiangsu Province China

## Abstract

Ischemic stroke remains a leading cause of mortality and long-term disability, with reperfusion injury contributing significantly to poor clinical outcomes. Microglia, the primary immune cells of the central nervous system, play a dual role in ischemic stroke by both exacerbating injury through neuroinflammation and supporting recovery through neuroprotection. This study aimed to explore the role of CD74, a gene upregulated in microglia following ischemia-reperfusion injury. Using single-cell RNA sequencing and bulk RNA sequencing, we identified CD74 as a potential target involved in microglial-mediated neuroinflammation. We observed a significant increase in CD74 expression in microglia following middle cerebral artery occlusion/reperfusion (MCAO/R), which correlated with pro-inflammatory cytokine production and neuroinflammation. Targeted knockdown of CD74 in microglia using CX3CR1Cre/ERT2 mice led to a reduction in infarct volume, inflammatory cytokine levels, and long-term neurological deficits. Behavioral tests showed improved motor coordination, sensory function, and exploratory behavior in CD74 knockdown mice. These results suggest that CD74 is a critical mediator of microglia-driven neuroinflammation, and targeting CD74 may represent a promising therapeutic strategy for reducing ischemic brain injury and promoting recovery after stroke.

**Supplementary Information:**

The online version contains supplementary material available at 10.1186/s13041-025-01197-8.

## Introduction

Ischemic stroke remains one of the leading causes of mortality and long-term disability worldwide [1]. The prevalence of ischemic stroke is expected to rise in the coming years, primarily driven by an aging population and increasing lifestyle-related risk factors [2]. Despite the advancements in treatment options, including endovascular thrombectomy and intravenous thrombolysis, which have become more widely adopted in recent years [3], reperfusion injury remains a significant contributor to poor clinical outcomes [4]. This ongoing challenge highlights the need for further research into improving post-stroke recovery and reducing the detrimental effects of reperfusion.

Microglia, the primary immune cells of the central nervous system, play a critical and multifaceted role in ischemic stroke and cerebral ischemia-reperfusion injury [5, 6]. Current research suggests that microglia exhibit dual functions during ischemic stroke, both contributing to injury and supporting recovery [7, 8]. On one hand, overactivation of microglia can exacerbate neuroinflammation and neuronal damage. This occurs through the release of neurotoxic substances such as reactive oxygen species (ROS), proteases, and pro-inflammatory cytokines, which lead to blood-brain barrier disruption and neuronal cell death [7]. Excessive microglial phagocytosis further contributes to delayed neuronal loss [9]. On the other hand, microglia also play a vital role in promoting recovery after ischemic injury. They help resolve inflammation, clear apoptotic cells, support neurogenesis, and remodel synapses, all of which contribute to neuroprotection [10, 11]. Additionally, microglia regulate the production of anti-inflammatory cytokines, such as transforming growth factor-β (TGF-β) and interleukin-10 (IL-10), which help restore homeostasis and prevent further neuronal damage [12, 13]. The functional outcome of microglia depends on their phenotypic state, which is influenced by their surrounding microenvironment [7, 14]. Following cerebral ischemia-reperfusion, microglia may adopt an pro-inflammatory phenotype that exacerbates injury, or an anti-inflammatory phenotype that supports repair and neuroprotection [15, 16]. However, the precise mechanisms governing their differential effects and the transition between these phenotypes remain an area of active investigation.

Single-cell RNA sequencing (scRNA-seq) provides a powerful tool for examining gene expression at the individual cell level, enabling the identification of cellular heterogeneity and diversity across various cell types and states under both normal and disease conditions [17]. This high-resolution transcriptional approach reveals complex cellular landscapes and enhances our understanding of disease mechanisms, offering new insights into potential therapeutic targets for ischemic stroke [18].

CD74 is a transmembrane glycoprotein primarily recognized for its role in stabilizing MHC II molecules during antigen processing. In autoimmune diseases such as rheumatoid arthritis and lupus, CD74 is upregulated, perpetuating inflammation via MIF-dependent pathways. Blocking this interaction has shown promise in reducing inflammatory responses [19, 20]. In neurodegenerative diseases like Alzheimer’s, CD74 is also upregulated in microglia and neurons, affecting amyloid-beta processing and tau phosphorylation, thereby contributing to neuroinflammation and degeneration. Targeting CD74 could help modulate inflammation and potentially slow Alzheimer’s progression [21]. However, the role of CD74 in ischemic stroke remains largely unexplored, underscoring the need for further research to clarify its mechanisms and therapeutic potential in this context.

In this study, we aimed to explore the dynamic transcriptomic profile of microglia following cerebral ischemia-reperfusion. To achieve this, we performed a comprehensive analysis using data from two public sources: scRNA-seq data from the ischemic hemisphere of mice one day post-middle cerebral artery occlusion/reperfusion (MCAO/R) [22] and bulk RNA sequencing data from sorted microglia from the ischemic hemisphere of mice three days post-MCAO/R [23]. Through this dual-dataset analysis, we identified Cd74 as a gene potentially involved in microglia-mediated neuroinflammation, which was upregulated following MCAO/R, suggesting its role in the inflammatory response triggered by ischemic injury. In this study, we observed a marked upregulation of Cd74 in microglia following cerebral ischemia-reperfusion injury. Targeted knockdown of Cd74 in microglia led to a reduction in neuroinflammation and decreased neuronal damage post-ischemia in mice.

## Materials and methods

### scRNA-seq data processing

10×Genomics scRNA-seq data (GEO accession GSE174574) [24] was analyzed in Python using the Scanpy package. Cells with over 20,000 UMIs, > 20% mitochondrial genes, or potential doublets were excluded. Batch effects were corrected with BBKNN, and the top 3000 variable genes were used for principal component analysis. Two-dimensional visualization was done via TSNE. Clusters were annotated based on known markers and merged when expressing the same marker set. Differentially expressed genes and volcano plots were generated using the SCP pipeline in R.

### Bulk RNA-Seq data processing

We will first read the scRNA-seq data using R to obtain 10x Genomics single-cell data from the GEO database (accession number GSE174574). Quality control will be performed on each sample, selecting cells with between 500 and 20,000 unique molecular identifiers (UMIs) and excluding those with a mitochondrial gene percentage greater than 25% for further analysis. We will utilize the DoubletFinder package (https://github.com/chris-mcginnis-ucsf/DoubletFinder) to remove doublets from the dataset.To correct for batch effects between groups, we will apply the Harmony algorithm (https://github.com/immunogenomics/harmony) for sample integration. Following data normalization and scaling, we will identify the 3,000 most variable genes in the dataset for principal component analysis. We will then use the FindClusters function for unsupervised clustering and visualize the results with UMAP.Clusters will be annotated based on previously identified marker genes and merged if they express the same set of marker genes. Clusters categorized as microglia will be extracted for differential expression analysis. All figures will be generated using built-in functions from the Seurat (v4.4) workflow and ggplot (v3.5.1) from the R tidyverse package.

### Ethics and animals

This study was approved by the Institutional Animal Care and Use Committee of the First Affiliated Hospital of Soochow University and followed NIH animal care standards. Wild-type C57BL6/J mice were obtained from the Chinese Academy of Sciences, and CX3CR1Cre/ERT2 mice from the Shanghai Nanfang Research Center. All mice were housed in pathogen-free conditions on a 12-hour light/dark cycle with food and water access. Male mice aged 8–12 weeks (20–25 g) were used for in vivo experiments.

### Establishment of MCAO/R model in mice

Mice were anesthetized with 3% isoflurane, maintained at 1.5%, and a midline neck incision was made to expose the right common (CCA), external (ECA), and internal carotid arteries (ICA). A 6–0 silicone-coated filament was inserted via the ECA into the ICA to occlude the middle cerebral artery (MCA), inducing ischemia for approximately 1.5 h, after which the filament was withdrawn to allow reperfusion [25]. Sham-operated mice underwent the same procedure without filament insertion.

### Cd74 knockdown in microglia via CX3CR1Cre/ERT2 mice and DIO-Sequence AAV

CX3CR1Cre/ERT2 mice (B6.129P2(C)-Cx3cr1tm2.1(cre/ERT2)Jung/J) were anesthetized with 3% isoflurane, maintained at 1.5%, and positioned in a stereotaxic frame (RWD Life Science, Shenzhen, China). A total of 1 µl of either rAAV-SFFV-DIO-mCherry-5’miR-30a-shRNA (Scramble) or rAAV-SFFV-DIO-mCherry-5’miR-30a-shRNA (CD74) virus (5 × 10^12 vg/µL) was injected into three target sites (2.0 mm lateral; +0.5, 0, -0.5 mm anterior-posterior to bregma; 1.5 mm depth) at 0.06 µl/min using a microsyringe pump. After injection, the syringe was left in place for 10 min. Fourteen days post-injection, tamoxifen (75 mg/kg, Selleck, China) was administered intraperitoneally for five consecutive days to activate Cre recombinase expression.

### 2, 3, 5-Triphenyltetrazolium chloride (TTC) staining

Mice were sacrificed 3 days post-MCAO/R, and brains were sectioned into 1-mm coronal slices. These were incubated in TTC solution (Jiancheng Biotech, Nanjing, China) for 15–30 min at 37 °C in the dark. Infarct volume, adjusted for edema, was calculated using ImageJ software with the formula: [(contralateral hemisphere volume - non-infarct area of ipsilateral hemisphere) / (contralateral hemisphere volume × 2)] × 100%.

### Western blot analysis

Brain tissues were homogenized in ice-cold lysis buffer (Beyotime, Shanghai, China) containing protease inhibitors. After centrifugation at 12,000 rpm for 20 min at 4 °C, protein concentrations in the supernatant were determined using a BCA assay kit (Beyotime). Proteins (20 µg per lane) were separated on a 12% SDS-PAGE gel, transferred to PVDF membranes (Millipore, USA), and blocked with 5% BSA (Biosharp, Hefei, China) for 1 h. Membranes were incubated overnight at 4 °C with the primary antibody (CD74, 1:200, #77274, Cell Signaling Technology, USA), followed by HRP-conjugated secondary antibody at room temperature for 1 h. Bands were detected by enhanced chemiluminescence, quantified with ImageJ (NIH), and β-tubulin was used as a loading control.

### Immunofluorescent analysis

Mouse brain tissue was perfused, fixed with paraformaldehyde, and dehydrated in a sucrose gradient for 2 days. The tissue was then frozen and sectioned into 20 μm coronal slices. Mounted sections were fixed in paraformaldehyde for 15 min, antigen-repaired for 15 min, and permeabilized with 0.1% Triton for 10 min. Sections were blocked with 5% BSA, incubated with primary antibodies overnight at 4 °C, followed by secondary antibodies at room temperature for 1 h. Nuclei were counterstained with DAPI, and fluorescence images were captured using an Olympus VS200 microscope (Olympus, Tokyo,apan).

### Enzyme-linked immunosorbent assay (ELISA)

Infarcted hemispheres from MCAO/R mice and corresponding hemispheres from sham controls were collected and homogenized in 0.9% saline 3 days post-MCAO/R. After centrifugation, the supernatant was analyzed for inflammatory cytokines using an ELISA kit (Bioswamp, Wuhan, China), with cytokine concentrations determined from OD values.

### Quantitative real-time PCR (qRT-PCR)

Total RNA was extracted from brain tissue using TRIzol (Invitrogen) following the manufacturer’s instructions. cDNA was synthesized with the High-Capacity RNA-to-cDNA Kit (Invitrogen). Target gene mRNA levels were quantified by real-time PCR using PowerUp SYBR Green Master Mix (Thermo Fisher Scientific, USA) and normalized to β-actin. Primer sequences were as follows: IL-1β (F: TCGCAGCAGCACATCAACAAGAG, R: AGGTCCACGGGAAAGACACAGG); TNF-α (F: ATGTCTCAGCCTCTTCTCATTC, R: GCTTGTCACTCGAATTTTGAGA); IL-6 (F: CTCCCAACAGACCTGTCTATAC, R: CCATTGCACAACTCTTTTCTCA); β-actin (F: GGCTGTATTCCCCTCCATCG, R: CCAGTTGGTAACAATGCCATGT).

### Rotarod test

Motor function was evaluated in mice at 3, 5, 7, 14, and 28 days following MCAO/R using the Rotarod test. Each group consisted of 10 mice, which were placed on a rotating cylinder that gradually accelerated. The time taken for the mice to fall off the rod was recorded as the fall latency. Prior to the surgery, the mice underwent five days of training. On the first two days, they were trained once daily on a slow-rotating rod (accelerating from 0 to 10 rpm over 30 s). On the third day, the mice were trained on both low-speed and high-speed rods, with acceleration from 4 to 40 rpm over 2 min. On the fourth day, the mice were trained twice on the high-speed setting, and on the fifth day, they were trained three times at high speed. The data collected on the fifth day were used as baseline measurements for comparison.

### Adhesive removal test

The adhesive removal test was employed to assess motor coordination deficits in mice following stroke. A piece of adhesive tape (0.4 × 0.3 cm²) was applied with consistent pressure to the medial aspect of the left forelimb. The mice were then placed into a transparent cage, and the time required for them to contact and remove the tape was measured, with a maximum duration of 120 s. Prior to surgery, the mice underwent training for five consecutive days, with three trials per day. Testing was conducted at 3, 5, 7, 14, and 28 days after the stroke model was established.

### Open field test

The open field test was employed to evaluate changes in exploratory behavior and neuropsychiatric function following cerebral ischemia in mice. The apparatus consisted of a plastic enclosure (40 × 50 × 40 cm) equipped with an automated video recording system. The floor of the box was divided into 4 × 4 grids, with the central four grids designated as the central zone. Mice were placed in the center of the field, and their spontaneous activity was recorded for 10 min. Key parameters, such as average speed and total distance traveled, were calculated. Additionally, the time spent in the central zone and the frequency of entries into this area were quantified. For acclimatization, each mouse was placed in an empty box for 15 min over five consecutive days. Testing began 24 h after the last acclimation session.

### Statistical analysis

Data were analyzed using GraphPad Prism (version 8.0, USA) and presented as means ± SEM. For comparisons between two groups, an unpaired two-tailed Student’s t-test was applied. For multiple group comparisons, one-way ANOVA with Tukey’s post hoc test was used. For two-way comparisons with two factors, two-way ANOVA followed by Bonferroni’s or Tukey’s post hoc analysis was performed. Statistical significance was set at *p* < 0.05.

## Results

### Microglial transcriptional profile altered following ischemic stroke

**Fig. 1:**
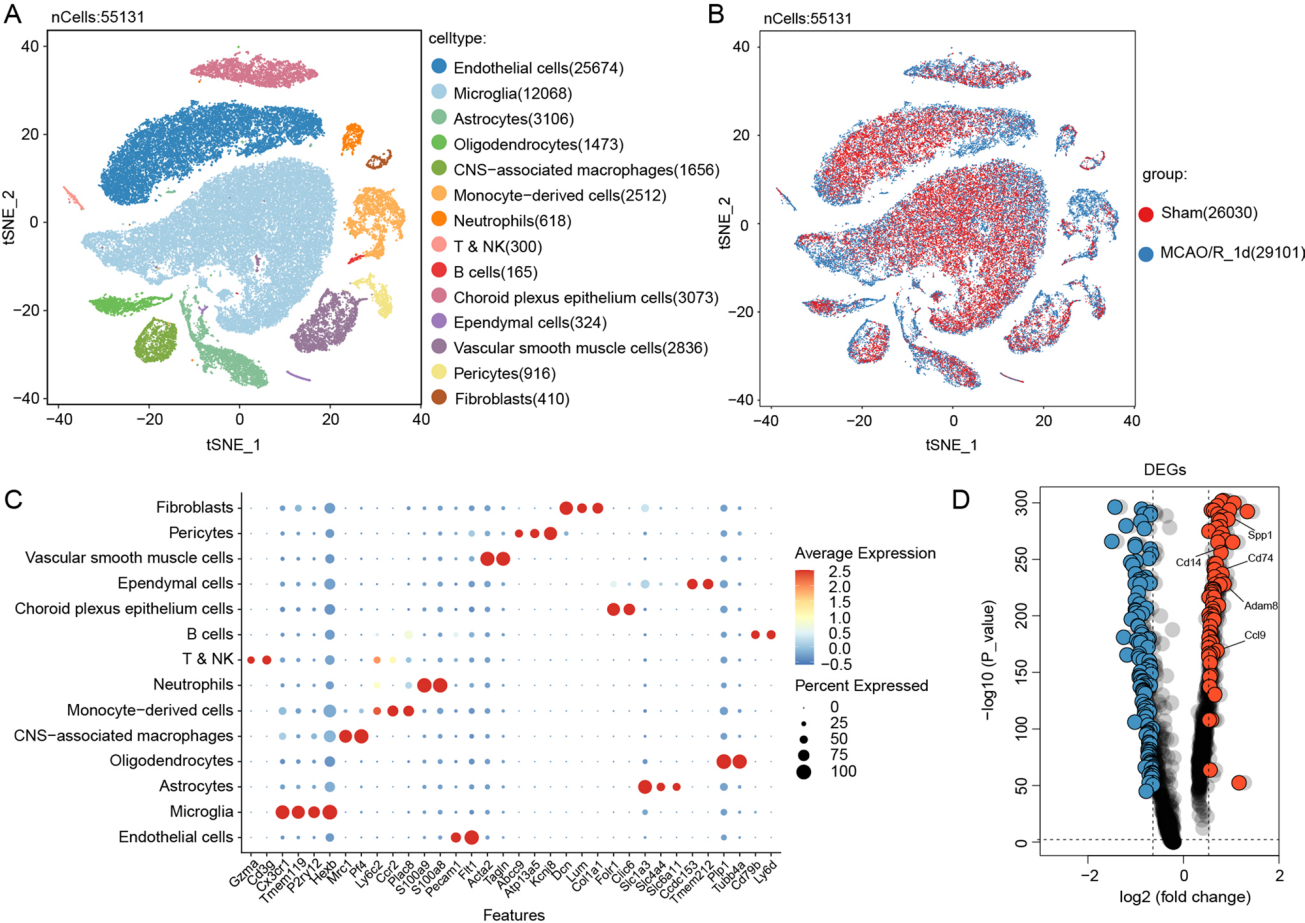
Single-Cell Gene Expression Profiling of Microglia 1 Day Post-MCAO/R in Mice. (**A**) t-SNE Plot: Single-cell data (GSE174574) from 1 day after MCAO/R and sham groups were clustered into distinct cell types. (**B**) Heatmap: Expression patterns of key marker genes across various cell types were visualized. (**C**) t-SNE Plot: Separation of MCAO/R and sham groups based on single-cell data was depicted. (**D**) Differentially Expressed Genes: Upregulated and downregulated genes in microglia from the MCAO/R group were identified, with color coding based on p-value (< 0.05) and|fold change| > 1.2

We analyzed the scRNA-seq dataset (GSE174574) from ischemic ipsilateral hemispheres of mice 24 h after MCAO/R, comprising 29,742 cells from the MCAO/R group and 26,191 from the Sham group. After integrating six samples to correct for batch effects, dimensionality reduction and unsupervised clustering revealed 16 distinct cell types (Fig. 1A-B), including endothelial cells, microglia, astrocytes, oligodendrocytes, ependymal cells, monocytes, and macrophages, with representative marker genes shown in a heatmap (Fig. 1C). Differential analysis of microglia from the MCAO/R and Sham groups identified significant gene expression changes, displayed in a volcano plot (Fig. 1D).

**Fig. 2:**
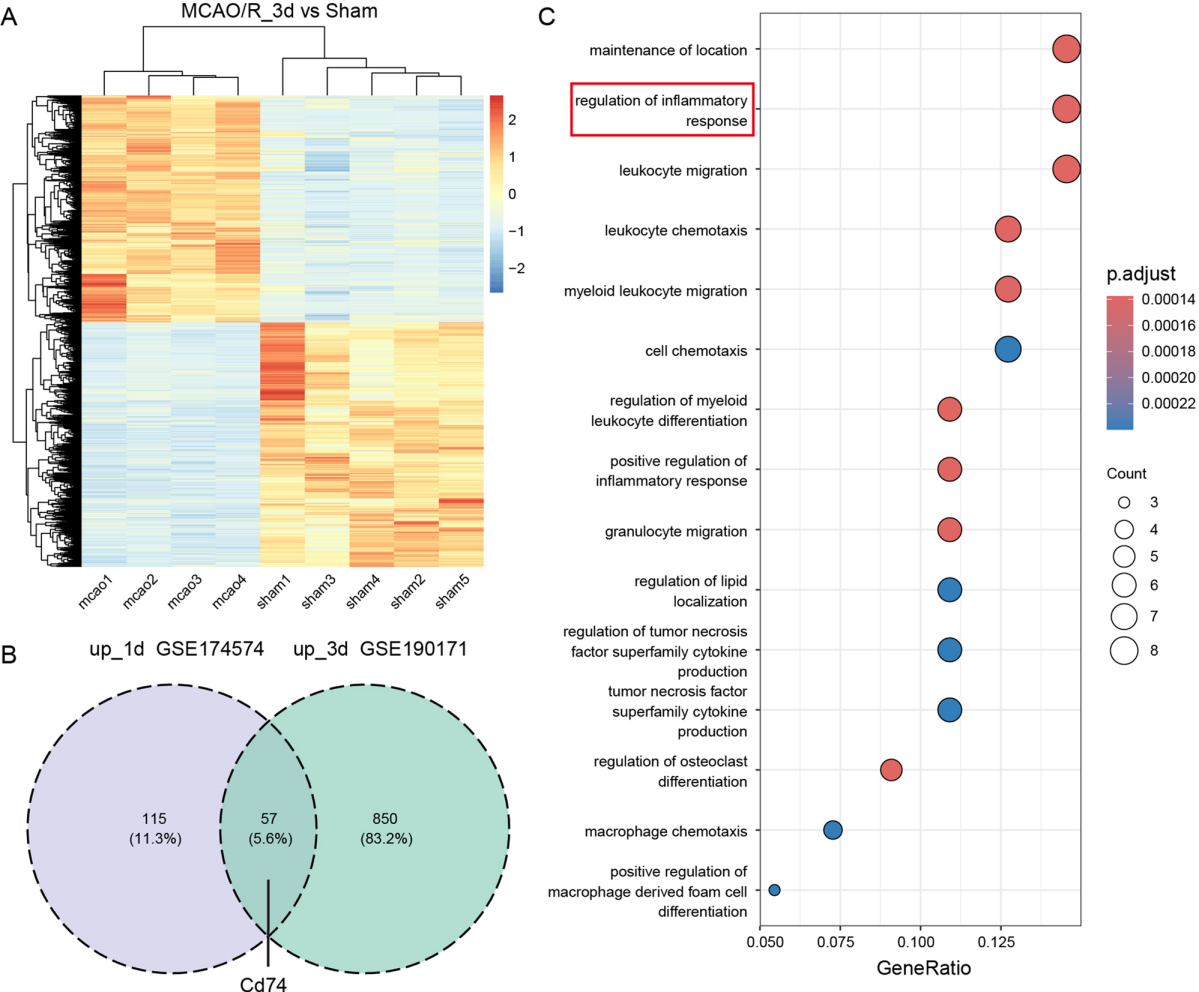
Gene Expression Analysis of Microglia 1 and 3 Days Post-MCAO/R in Mice Using Integrated Bulk RNA-seq Data. (**A**) Heatmap: Differential gene expression in microglia from the MCAO/R 3-day group was displayed compared to the Sham group using bulk RNA-seq data (GSE190171) obtained 1 day after MCAO/R. Genes were selected based on an adjusted p-value < 0.05 and|fold change| > 1.5. (**B**) Venn Diagram: Overlap between upregulated differential genes in microglia identified from single-cell (GSE174574) and bulk RNA-seq (GSE190171) data was illustrated. (**C**) GO Enrichment Analysis: Results of GO enrichment for the shared upregulated genes in microglia derived from both single-cell (GSE174574) and bulk RNA-seq (GSE190171) data were shown

To identify key genes associated with microglia after ischemic stroke, we further analyzed the bulk RNA-seq dataset (GSE190171) from flow-sorted microglia 3 days post-MCAO/R. Differential gene expression analysis (*p* < 0.05, log2FC > 0.585) was conducted, with the heatmap of differentially expressed genes shown (Fig. 2A). We intersected the differentially expressed genes from both the scRNA-seq (GSE174574) and bulk RNA-seq (GSE190171) datasets, identifying 57 overlapping genes (Fig. 2B). GO enrichment analysis revealed key pathways, including regulation of inflammatory responses, leukocyte migration, and myeloid leukocyte migration (Fig. 2C). In the GO analysis of differentially expressed genes in the neuroinflammation pathway, we identified CD74 as the most significantly different gene. Notably, CD74, a gene not previously studied in cerebral ischemia, was identified as a potential target for further investigation into its role and underlying mechanisms in ischemic stroke.

### CD74 expression in microglia was increased following MCAO/R

**Fig. 3:**
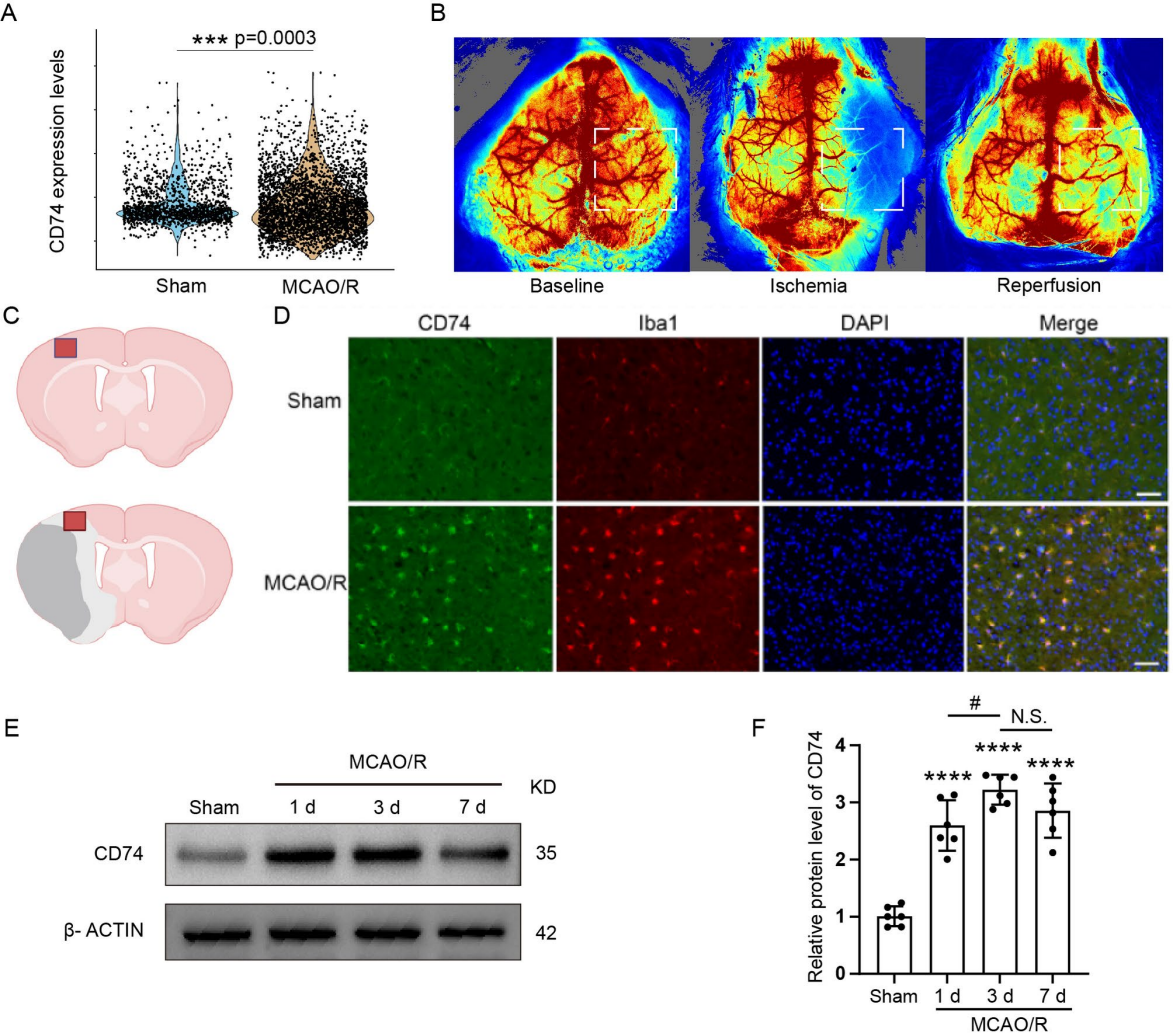
ElevatedCD74 Expression in Microglia After MCAO/R. (**A**) Violin Plot: Comparison of CD74 expression levels in microglia from MCAO/R and Sham groups 1 day post-modeling was presented. (**B**) Laser Speckle Imaging: Changes in cerebral blood flow were visualized before surgery (baseline), during ischemia (MCAO), and 10 min after reperfusion (MCAO/R). (**C, D**) Immunofluorescence: CD74 (green) and Iba1 (red) expression was detected in the cortical penumbra 3 days after MCAO/R. The region for microscopic analysis is marked by a hollow square in the left panel; scale bar = 50 μm. (**E, F**) Western Blot Analysis: CD74 protein expression levels were evaluated in the peri-infarct region of sham and ischemic mice at 1, 3, and 7 days post-operation. Data were presented as means ± SEM. ***p < 0.001 vs. Sham group; #*p*< 0.05, MCAO/R 3 d vs. MCAO/R 1 d group; ns, no significant difference between MCAO/R 7 d and MCAO/R 3 d groups

Based on the integrated transcriptomic analysis, we hypothesized that CD74 in microglia is significantly modulated after MCAO/R and may contribute to ischemia-reperfusion injury. scRNA-seq data indicated a substantial increase in CD74 mRNA levels in microglia at 1 days following MCAO/R (Fig. 3A). To examine the corresponding protein expression, we performed immunofluorescence assays in the MCAO/R mouse model. The fluorescence images revealed negligible CD74 expression in microglia from the Sham group, while a notable upregulation of CD74 was observed in the cortical penumbra following MCAO/R, primarily localized to microglia (Fig. 3B-D). These CD74-positive microglia displayed an activated morphology (Fig. S3A). Furthermore, Western blot analysis of penumbral tissue confirmed a significant elevation of CD74 protein levels in the MCAO/R group, with the most pronounced increase occurring at 3 days post-surgery (Fig. 3E-F).

### Knockdown of CD74 in microglia attenuated brain injury following cerebral ischemia-reperfusion

**Fig. 4:**
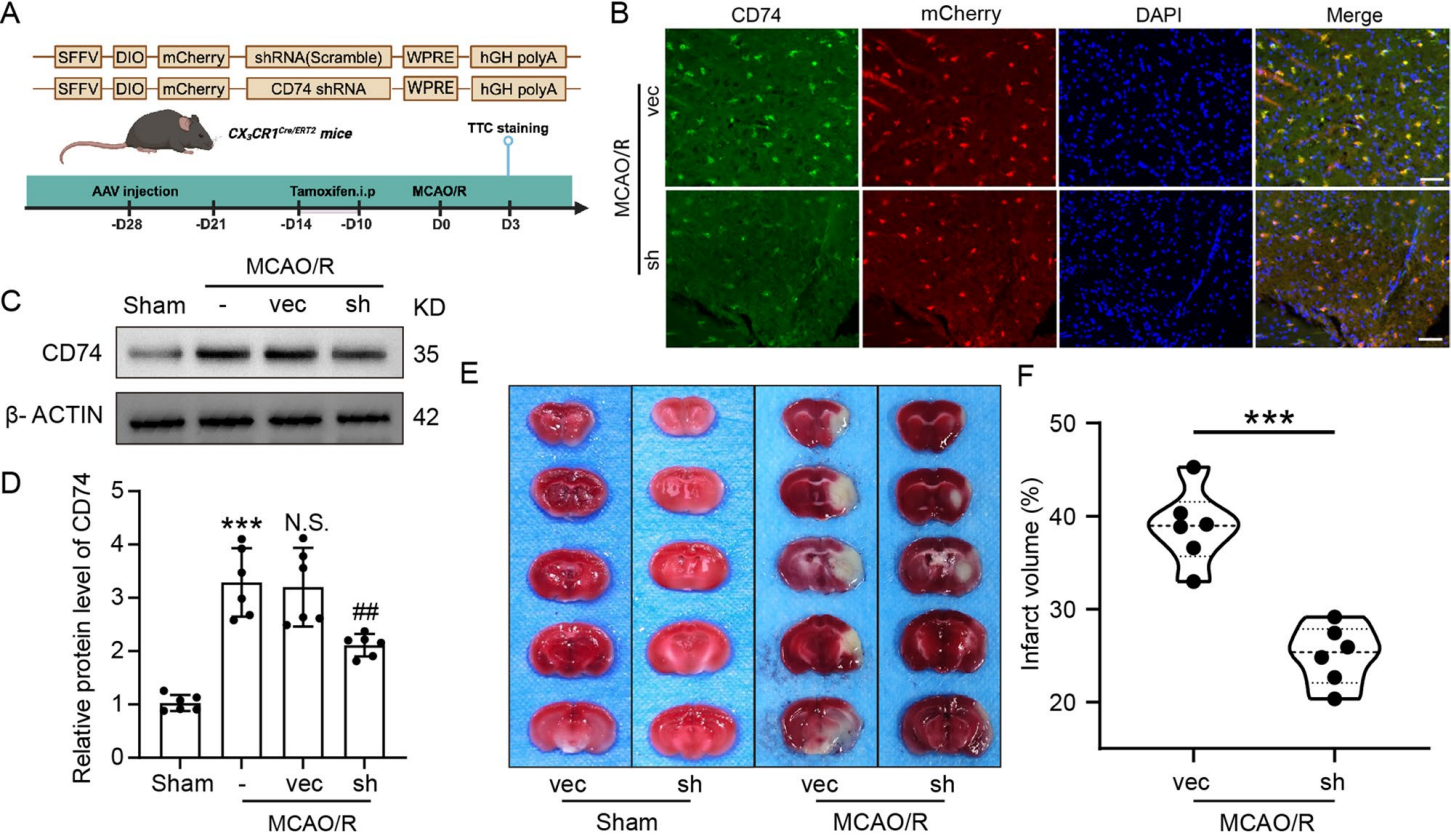
Microglia-Specific CD74 Knockdown Mitigates Brain Injury 3 Days Post-MCAO/R in Mice. (**A**) Experimental Design: A schematic of the strategy for microglia-specific CD74 knockdown in mice was presented. (**B**) Immunofluorescence: CD74 knockdown in microglia 3 days after MCAO/R was validated by CD74 (green) and Iba1 (red) staining; scale bar = 50 μm. (**C, D**) Western Blot Analysis: CD74 knockdown in the peri-infarct region 3 days after MCAO/R was validated. Data were shown as means ± SEM. ***p < 0.001 vs. Sham group; ##*p* < 0.01 vs. MCAO/R + vec group; N.S., no significant difference vs. MCAO/R group (*n* = 6). (**E, F**) TTC Staining: Cerebral infarct volume in mice with microglia-specific CD74 knockdown following MCAO/R was assessed. Data were presented as means ± SEM. **p < 0.01 vs. MCAO/R + vec group (*n* = 6)

To further explore the role of CD74 in microglia during ischemia-reperfusion injury, we employed CX3CR1 ^Cre/ERT2^ mice along with adeno-associated viruses carrying a DIO sequence of small interfering RNA to achieve targeted knockdown of CD74 specifically in microglia (Fig. 4A, Fig. S2A). Immunofluorescence analysis revealed a marked reduction in CD74 fluorescence intensity in the CD74 knockdown group compared to the Vector group, confirming successful knockdown of CD74 expression (Fig. 4B). Western blot analysis of penumbral tissue further corroborated these findings, showing significantly reduced CD74 levels in the sh-CD74 group (Fig. 4C-D).

To assess the functional consequences of CD74 knockdown, we performed TTC staining to evaluate infarct area changes. The results demonstrated that CD74 knockdown in microglia led to a significant reduction in infarct volume after MCAO/R (Fig. 4E-F), highlighting the involvement of CD74 in the pathophysiology of ischemia-reperfusion injury. Collectively, these findings suggest that targeted silencing of CD74 in microglia can mitigate brain injury following cerebral ischemia-reperfusion.

### Knockdown of CD74 in microglia preserved long-term neurological function following cerebral ischemia-reperfusion

**Fig. 5:**
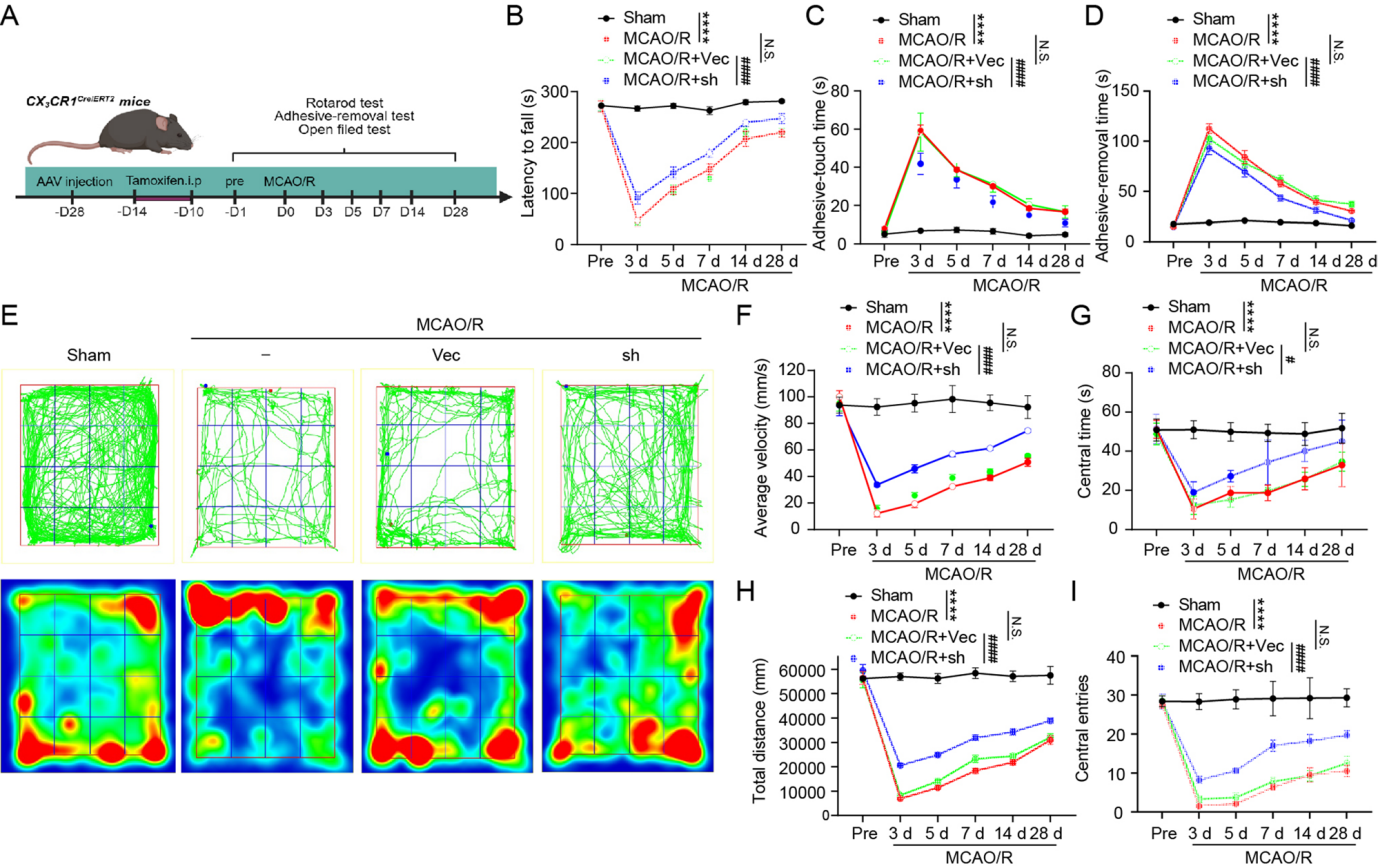
Microglia-Specific CD74 Knockdown Improves Long-Term Neurological Function in Mice After MCAO/R. (**A**) Experimental Procedure: A schematic representation of the study design was provided. (**B**) Rotarod Test: Latency to fall was measured before and up to 28 days post-MCAO/R. *n* = 10. (**C**) Adhesive Removal Test: Time to detect adhesive tapes was measured before and up to 28 days post-MCAO/R. *n* = 10. (**D**) Adhesive Removal Test: Time to remove adhesive tapes was measured before and up to 28 days post-MCAO/R. *n* = 10. (**E**) Open Field Test: Representative locomotion trajectories and heatmaps for different groups of mice were shown. (**F**-**G**) Open Field Test: Average speed and total distance traveled by different groups of mice before and up to 28 days after MCAO/R were calculated. *n* = 10. (**H**-**I**) Open Field Test: Time spent in the center region and number of entries into the center were assessed before and up to 28 days post-MCAO/R. *n* = 10. Results were shown as means ± SEM. ****p < 0.0001, Sham vs. MCAO/R group; #*p* < 0.05, ####*p* < 0.0001, MCAO/R + sh vs. MCAO/R + Vec group; ns, no significant difference, MCAO/R vs. MCAO/R + Vec group

To evaluate the effects of CD74 knockdown in microglia on long-term neurological recovery following cerebral ischemia-reperfusion, a series of behavioral assessments were performed (Fig. 5A). The rotarod test revealed that CD74 knockdown prolonged the latency to fall in mice after MCAO/R, indicating a reduction in motor deficits (Fig. 5B). The adhesive removal test further demonstrated that, compared to the MCAO/R + Vec group, both the contact and removal times of the adhesive labels were significantly shortened in the MCAO/R + sh-CD74 group, suggesting an improvement in sensory and motor coordination (Fig. 5C–D).

Exploratory behavior and anxiety were assessed using the open field test. Movement trajectory and density plots showed that, relative to the Sham group, MCAO/R mice exhibited reduced mobility and a more restricted activity area. However, in the MCAO/R + sh-CD74 group, mice displayed increased movement velocity and distance, indicating enhanced exploratory behavior and vitality following CD74 knockdown in microglia/macrophages (Fig. 5E–G). Additionally, an increase in the number of entries into the central zone and a longer duration spent there suggested a reduction in anxiety in the MCAO/R + sh-CD74 mice (Fig. 5H, I). These results collectively indicate that CD74 knockdown in microglia significantly improved long-term neurological function after cerebral ischemia-reperfusion.

### Microglial CD74 is involved in inflammatory cytokine production following cerebral ischemia-reperfusion

**Fig. 6:**
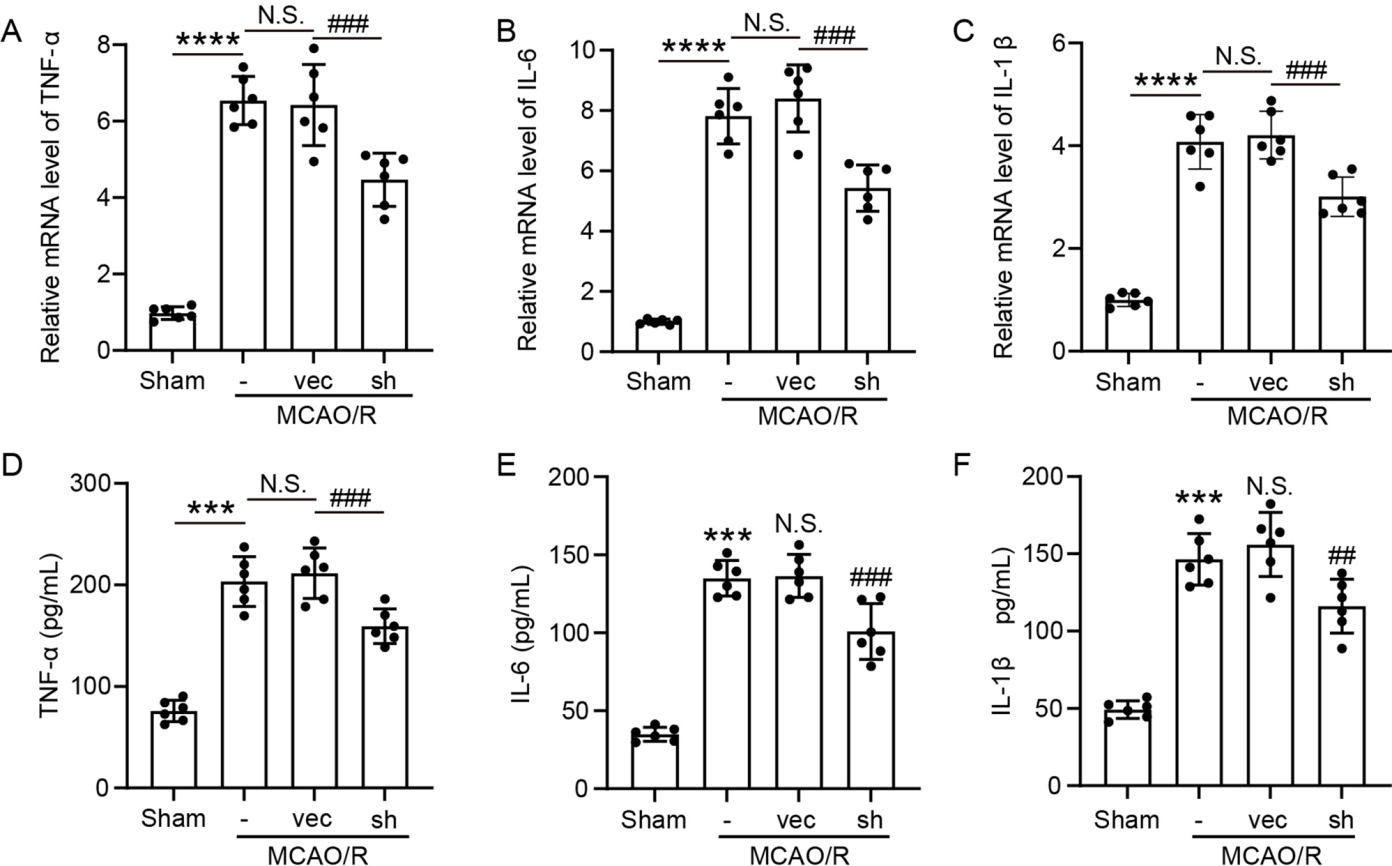
Microglia-Specific CD74 Knockdown Reduces Inflammatory Cytokine Production 3 Days Post-MCAO/R in Mice. (**A**-**C**) qRT-PCR: IL-1β, IL-6, and TNF-α mRNA levels in peri-infarct brain tissues of mice 3 days post-MCAO/R were measured. Data were presented as means ± SEM. ^###^*p* < 0.001, ****p < 0.0001; ns, no significant difference (*n* = 6). (**D**-**F**) ELISA: Protein levels of IL-1β, IL-6, and TNF-α in peri-infarct brain tissues 3 days post-MCAO/R were measured. Data were shown as means ± SEM. ^###^*p* < 0.001, ^***^p < 0.001, ^**^p < 0.005; ns, no significant difference (*n* = 6)

Given that CD74 expression was nearly undetectable in microglia from the Sham group but markedly elevated in microglia from the MCAO/R group, where microglia exhibited a pro-inflammatory phenotype (Fig. 6A-C), we further examined the effect of CD74 knockdown in microglia on the levels of key inflammatory factors following cerebral ischemia-reperfusion. ELISA results revealed significant increases in the pro-inflammatory cytokines TNF-α, IL-1β, and IL-6 in brain tissue after ischemic stroke (Fig. 6D-F). Notably, specific knockdown of CD74 in microglia resulted in a substantial reduction in the expression of these inflammatory mediators.

## Discussion

This study provides strong evidence for the role of CD74 in microglia-mediated neuroinflammation following cerebral ischemia-reperfusion injury. Through single-cell RNA sequencing (scRNA-seq) and bulk RNA sequencing, we identified CD74 as a gene significantly upregulated in microglia after middle cerebral artery occlusion/reperfusion (MCAO/R). Additionally, we observed the upregulation of several classical pro-inflammatory genes; for instance, the increased expression of Spp1 may promote the recruitment and activation of inflammatory cells, while the activation of Cd14 leads to the release of pro-inflammatory mediators. Our findings reveal a correlation between CD74 expression, microglial activation, pro-inflammatory cytokine production, and the exacerbation of ischemic injury, highlighting its importance in the pathophysiology of ischemic stroke.

Our integrated transcriptomic analysis demonstrated that CD74 was prominently expressed in microglia during ischemia-reperfusion injury and colocalized with activated microglia in the cortical penumbra. These observations align with prior studies in autoimmune and neurodegenerative diseases [26], where CD74 is implicated in inflammation and pathological progression. However, its specific role in ischemic stroke has remained largely unexplored. By employing targeted knockdown of CD74 in microglia using CX3CR1^Cre/ERT2^ mice and adeno-associated viruses, we demonstrated that CD74 suppression mitigated infarct volume, reduced inflammatory cytokine production, and preserved long-term neurological function. This underscores the detrimental role of microglial CD74 in ischemia-reperfusion injury.

Microglial activation following ischemia-reperfusion is a complex and dynamic process [7]. While microglia play a dual role in neuroinflammation and recovery [27], our study suggests that CD74 may tip this balance toward a pro-inflammatory phenotype, driving neurotoxicity. Specifically, CD74 knockdown reduced the expression of TNF-α, IL-1β, and IL-6, three critical inflammatory cytokines that contribute to blood-brain barrier disruption, neuronal injury, and poor neurological outcomes. These findings indicate that targeting CD74 may serve as a therapeutic strategy to modulate microglial activation and alleviate the detrimental effects of ischemic stroke.

In addition to reducing infarct size and inflammatory responses, CD74 knockdown significantly improved long-term neurological recovery. Behavioral assessments, including the rotarod test, adhesive removal test, and open field test, revealed that mice with CD74 knockdown exhibited enhanced motor coordination, sensory function, and exploratory behavior compared to controls. This highlights the potential of CD74-targeted interventions in promoting both acute recovery following ischemic stroke.

Despite these promising findings, our study has limitations. First, while the use of mouse models allows for mechanistic insights, the translation of these findings to human ischemic stroke remains uncertain. Future studies should validate the role of CD74 in human microglia and explore its therapeutic potential in clinical settings. Second, This study focused on the effects of CD74 knockdown on infarct volume and short-term functional prognosis, and its downstream molecular mechanisms (such as specific signaling pathways and cell interaction networks) still need to be further analyzed by conditional gene knockout, single cell sequencing and other techniques. These directions will be the focus of our follow-up research. Understanding these pathways could uncover additional therapeutic targets and refine intervention strategies.

In conclusion, this study identifies CD74 as a critical mediator of microglia-driven neuroinflammation and ischemic brain injury. By targeting CD74, we observed significant reductions in infarct size, inflammation, and long-term functional deficits, providing a strong rationale for further exploration of CD74 as a therapeutic target in ischemic stroke. These findings contribute to the growing body of evidence supporting the modulation of microglial function as a promising strategy for mitigating the deleterious effects of ischemia-reperfusion injury.

## Electronic supplementary material

Below is the link to the electronic supplementary material.

Supplementary Material 1

Supplementary Material 2

Supplementary Material 3

## Data Availability

No datasets were generated or analysed during the current study.

## Declarations

### Artificial intelligence (AI)-Assisted technology

During the preparation of this work the author(s) used Chatgpt (versions 4.0) in order to proofread the manuscript for spelling and grammar. After using this tool, the author(s) reviewed and edited the content as needed and take(s) full responsibility for the content of the publication.
