# Supplementary figures and images for: Targeting CD74 in microglia to modulate experimental cerebral ischemia and reperfusion injury: insights from Single-Cell and bulk transcriptomics
Source: Mol Brain. 2025 May 21;18:46. doi: 10.1186/s13041-025-01197-8 (PMC12096678; doi:10.1186/s13041-025-01197-8)

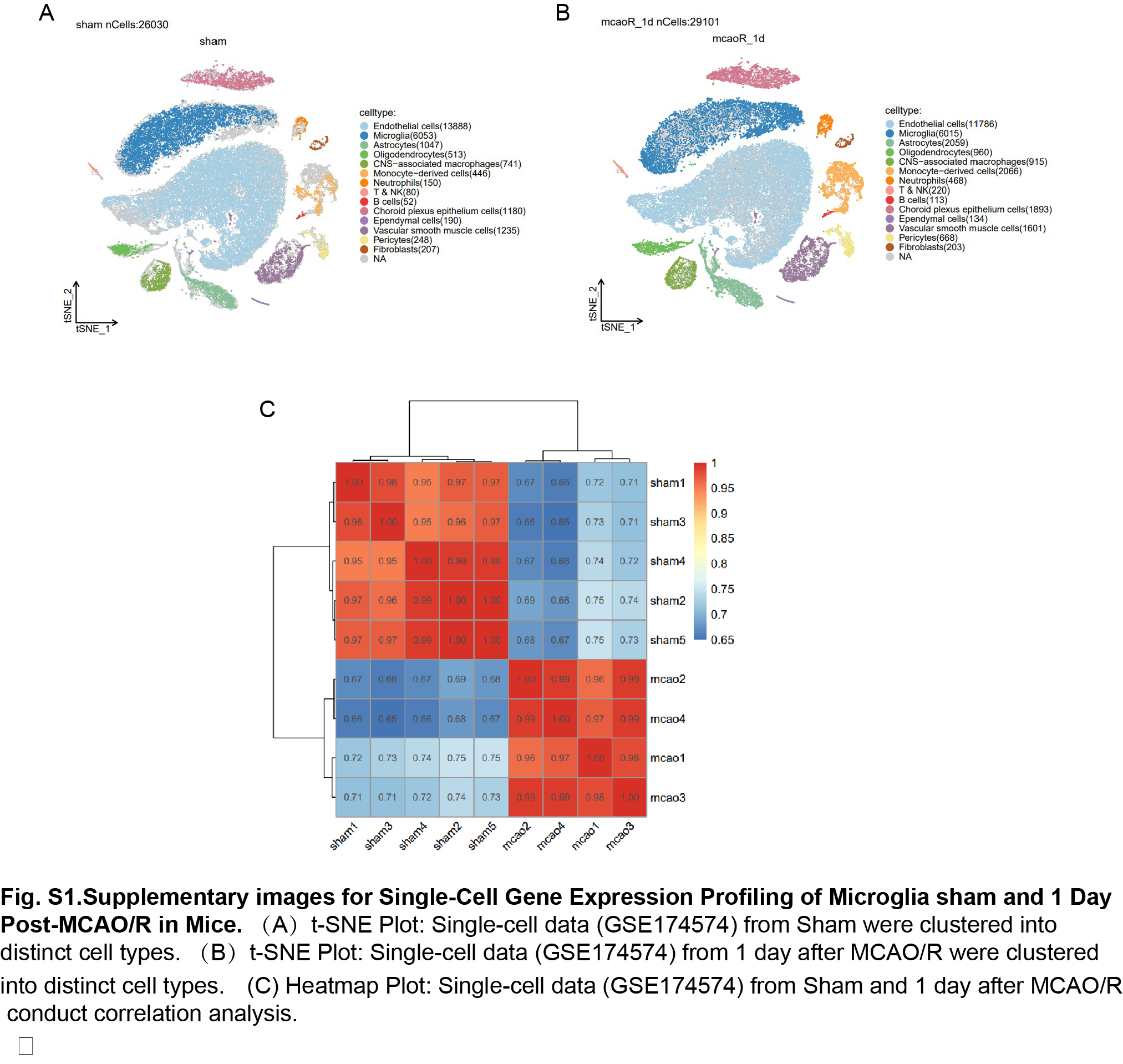

Supplement: Supplementary file 1 — Supplementary Material 1 [file 13041_2025_1197_MOESM1_ESM.jpg]

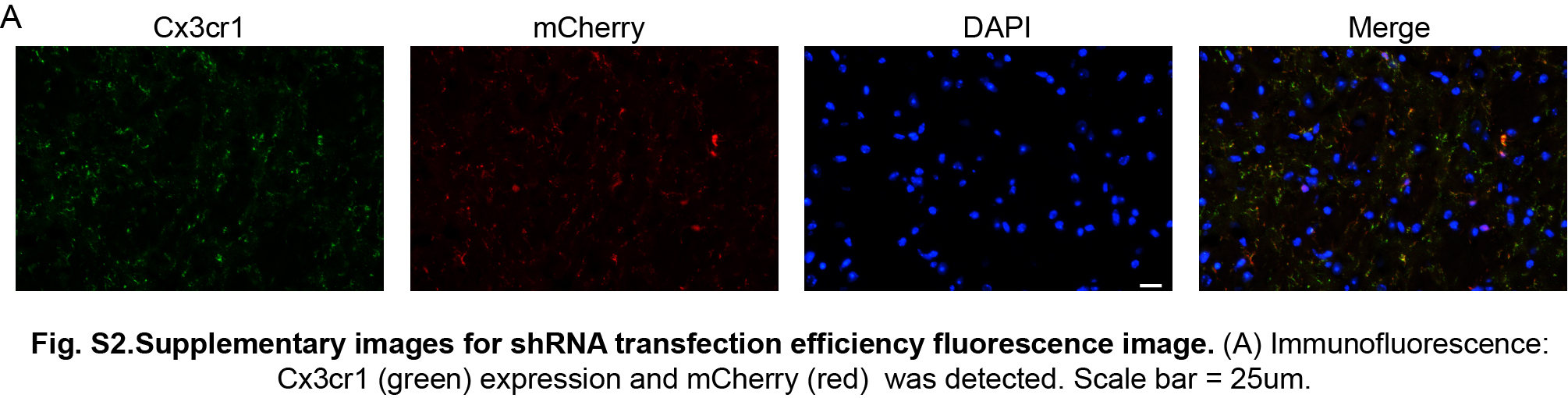

Supplement: Supplementary file 2 — Supplementary Material 2 [file 13041_2025_1197_MOESM2_ESM.jpg]

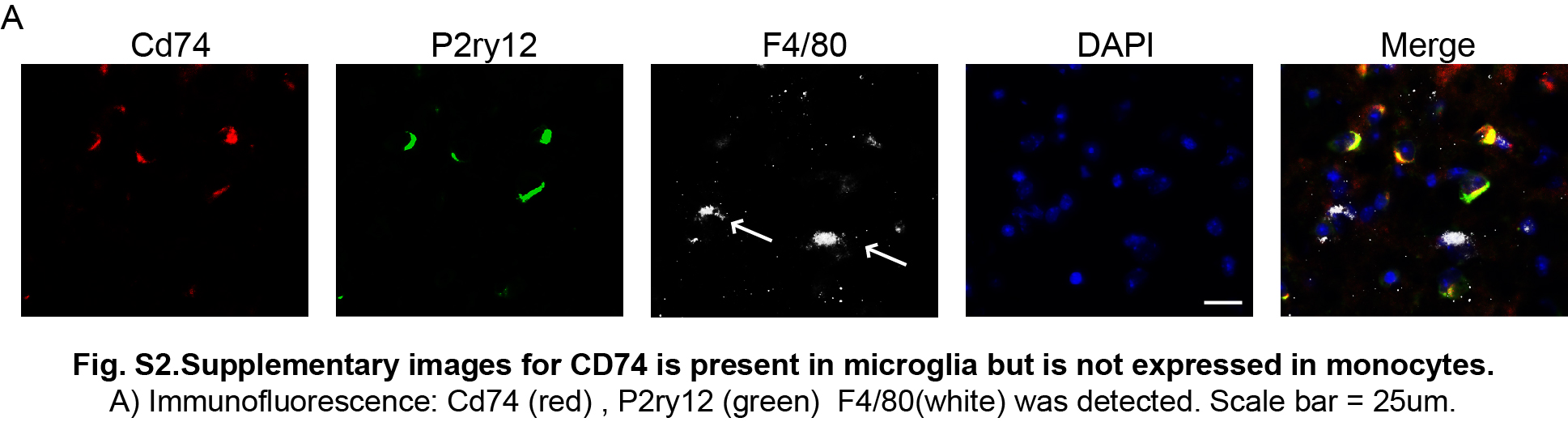

Supplement: Supplementary file 3 — Supplementary Material 3 [file 13041_2025_1197_MOESM3_ESM.jpg]
